# Glucosamine Enhances TRAIL-Induced Apoptosis in the Prostate Cancer Cell Line DU145

**DOI:** 10.3390/medicines6040104

**Published:** 2019-10-15

**Authors:** Chao Sun, Viktor Chesnokov, Garrett Larson, Keiichi Itakura

**Affiliations:** Department of Molecular and Cellular Biology, Beckman Research Institute of City of Hope, Duarte, CA 91010, USA

**Keywords:** glucosamine, ER stress, DR5, TRAIL, apoptosis, cancer, DU145

## Abstract

**Background**: Tumor necrosis factor (TNF)-related apoptosis-inducing ligand (TRAIL) selectively kills tumor cells in cancer patients. However, patients often develop TRAIL resistance; thus, agents that can sensitize cells to TRAIL therapy would be beneficial clinically. **Methods**: Immunoblotting, flow cytometry, confocal microscopy, qPCR and caspase 8 activity assays were used to investigate whether glucosamine (GlcN) can sensitize cancer cells to TRAIL thereby enhancing apoptosis and potentially improving clinical response. **Results**: GlcN sensitized DU145 cells to TRAIL-induced apoptosis but did not increase death receptor 5 (DR5) cell surface expression. Once treated, these cells responded to TRAIL-induced apoptosis through both extrinsic and intrinsic apoptotic pathways as evidenced by the cleavage of both caspases 8 and 9. The combination of GlcN and TRAIL suppressed the expression of key anti-apoptotic factors cFLIP, BCL-X_L_, MCL-1 and XIAP and translocated BAK to the mitochondrial outer membrane thereby facilitating cytochrome C and SMAC release. In addition to the activation of apoptotic pathways, TRAIL-mediated inflammatory responses were attenuated by GlcN pretreatment reducing nuclear NF-kB levels and the expression of downstream target genes *IL-6* and *IL-8*. **Conclusions**: GlcN/TRAIL combination could be a promising strategy for treating cancers by overcoming TRAIL resistance and abrogating TRAIL-induced inflammation.

## 1. Introduction

Tumor necrosis factor (TNF)-related apoptosis-inducing ligand (TRAIL) is a member of the TNF family that triggers apoptosis in cancer cells through activation of death domain-containing TRAIL receptors, TRAIL-R1 (DR4) and TRAIL-R2 (DR5) [1]. After binding to DR4 or DR5, TRAIL mainly activates the extrinsic apoptosis pathway via caspase mediators [2]. TRAIL was initially considered an attractive anticancer agent because of its ability to selectively induce cancer cell-specific apoptosis in multiple cell lines; however, clinical trials with TRAIL or anti-TRAIL receptor antibodies yielded disappointing results [3,4,5,6,7]. Subsequently, it was determined that some cancer cells were resistant to TRAIL monotherapy through a variety of mechanisms thereby evading cell death. To overcome this resistance, a variety of sensitizing agents have been explored, several of which increased sensitivity of cancer cells to TRAIL-induced apoptosis. Often the increased sensitivity of these cells to TRAIL was associated with augmented *DR5* expression [8,9,10,11].

DR5 is a type I transmembrane receptor with a cytoplasmic death domain which recruits apoptosis signaling factors for the induction of cell death. However, DR5 is either expressed at low levels or not expressed in many cancer cells. The expression of *DR5* is transcriptionally up-regulated by CCAAT-enhancer-binding protein homologous protein (CHOP) which is induced under endoplasmic reticulum (ER) stress. This suggests that agents which increase ER stress may also increase TRAIL sensitivity [12]. Tunicamycin, an inhibitor of protein N-glycosylation [13], triggers ER stress via the accumulation of proteins deficient in N-glycosylation and enhances TRAIL-induced apoptosis in human prostate cancer cells [9]. Although tunicamycin is a promising candidate for combination therapy with TRAIL, severe toxicity limits its application in humans [14]. Like tunicamycin, GlcN inhibits N-glycosylation of proteins and induces ER stress but has low toxicity and is efficiently transported into tumor cells [15,16]. Although reduced DR4 and DR5 expression is often observed in cancer cells, additional mechanisms likely contribute to TRAIL resistance. Cancer cells may overexpress a host of downstream anti-apoptotic regulators. These include c-FLIP, an inhibitor of caspase 8 cleavage/activation reaction, some members of the anti-apoptotic BCL-2 family, and IAP family members, inhibitors of caspases 3 and 9 [2,17,18,19,20,21].

In addition to apoptosis, TRAIL promotes tumor growth mainly through the transcriptional factor NF-kB which plays a role in inflammation, immune response and cell proliferation [22,23,24]. NF-kB dysregulation can lead to the development of multiple diseases, including rheumatoid arthritis, inflammatory bowel diseases and cancer [25]. NF-kB suppression can block the progression of multiple human tumors [26,27]. This suggests that inhibition of TRAIL-induced NF-kB signal transducing pathway could enhance TRAIL-induced apoptosis in cancer cells [28,29].

We determined that GlcN up-regulated the expression of DR5; however, it did not increase cell surface expression suggesting that GlcN mediates apoptosis through alternative mechanisms. The combination of GlcN and TRAIL (GlcN/TRAIL) increased the activities of caspases 8, 9 and 3, further enhancing apoptosis over TRAIL alone in cancer cell lines sensitive to GlcN-induced deglycosylation such as DU145 prostate cancer cells. Mechanistic studies revealed that GlcN/TRAIL stimulated both the extrinsic and intrinsic apoptotic pathways. Collectively this led to a decrease in c-FLIP, BCL-X_L_, MCL-1, cIAP-1 and XIAP expression and translocated BAK increasing the permeability of the mitochondrial outer membrane leading to increased cytochrome C and SMAC release. Pretreatment of cells with GlcN also reduced TRAIL-induced nuclear NF-kB levels. Our data indicated that caspase 8 activation is required for apoptosis and the targeted suppression with a caspase 8 specific inhibitor reversed apoptosis caused by the GlcN/TRAIL combination. These data suggest that GlcN might be a promising candidate for combined anti-cancer therapy with TRAIL.

## 2. Materials and Methods

### 2.1. Cell Culture, Chemical Compounds and Biological Reagents

Human prostate cancer cell lines DU145, PC3 and C4-B cells along with HeLa cervical cancer cells were obtained from the American Type Culture Collection (Manassas, VA, USA). Cells were cultured in RPMI 1640 medium supplemented with glutamine, essential amino acids (Irvine Scientific, Santa Ana, CA, USA), 10% fetal bovine serum (Omega Scientific Inc., Tarzana, CA, USA) and antibiotics (100 U/mL penicillin G and 100 µg/mL streptomycin, Mediatech Inc., Manassas, VA, USA). Cells were incubated at 37 °C in 5% CO_2_ and passaged when 70% confluent using trypsin/EDTA disaggregation. For the combination GlcN and TRAIL treatment, cells were seeded in RPMI 1640 medium, incubated with 2mM GlcN (glucosamine hydrochloride, Sigma, St. Louis, MO, USA) for 24 h, then treated with 50 ng/mL TRAIL for 8 h. RPMI 1640 medium was purchased from Irvine Scientific (Santa Ana, CA, USA); RIPA buffer was purchased from G-Biosciences (Saint Louis, MO, USA). Protease and phosphatase inhibitor cocktail and BCA protein assay kit were purchased from Thermo Scientific (Rockford, IL, USA). Amersham ECL prime detection reagent was purchased from GE Healthcare. TRAIL (T5694) was purchased from Sigma-Aldrich Corporation (Saint Louis, MO, USA). FcR blocking reagent (130-059-901) was purchased from Miltenyl Biotec (Auburn, CA). Z-IETD-FMK was obtained from Fisher Scientific (Waltham, MA, USA). When Z-IETD-FMK was used, it was added to samples 1.5 h before TRAIL treatment at a concentration of 50 µM. Antibodies were obtained from Cell Signaling Technology (Beverly, MA, USA): anti-mouse IgG HRP-linked (7076), anti-rabbit IgG HRP-linked (7074), DR4 (42533), DR5 (8074), pan-actin (12748), phospho-eIF2a (3398), ATF4 (11815), CHOP (2895), XBP1s (12782), Bip/Grp 78 (3183), BID (2002), cFLIP (56343), BCL-X_L_ (2764), MCL-1 (39224), XIAP (2042), cIAP-1 (7065), phospho-NF-kB (3033), cleaved caspase 8 (9496), cleaved caspase 9 (7327), cleaved caspase 3 (9664), cleaved PARP (5625), EGFR (2232) and GAPDH (97166). ATF6 (ab122897) and TOMM20 (ab186734) were from AbCam (Cambridge, MA, USA). DR4-PE (FAB 347P), DR5-PE (FAB 6311P), RIP1 (MAB3585), mouse IgG1-PE (IC002P) and mouse IgG2B-PE (IC004P) were from R&D systems (Minneapolis, MN, USA). BAK (AM03) was from Millipore and TOPO-1 (TG2012-2) was from TopoGEN (Buena Vista, CO, USA).

### 2.2. Immunoblot Analysis

Cells grown in six-well plates were washed with PBS and then lysed with RIPA buffer supplemented with protease and phosphatase inhibitor for 15 min at 4 °C. Lysates were vortexed for 15 sec to shear DNA and subsequently centrifuged at 12,000 × *g* for 10 min at 4 °C. Protein concentration was quantified by the BCA protein assay kit and extracts were used immediately for SDS-PAGE or aliquoted and stored at −80 °C. Protein extracts were separated on 8% or 4–20% gradient SDS-PAGE gels and electrophoretically transferred onto polyvinylidene difluoride (PVDF) membranes (Trans-Turbo Blot, Bio-Rad Laboratories, Hercules, CA, USA). Following blocking, targets were detected using primary antibodies and horseradish peroxidase (HRP)-conjugated secondary antibodies and chemiluminescent Amersham ECL prime reagent. Protein size was determined by Precision Plus protein standards from Bio-Rad Laboratories. Immunoblotting densitometry was quantified by publicly available free ImageJ 1.45S software. (NIH, Bethesda, MD, USA).

### 2.3. Quantitative RT PCR

Total RNA was extracted using Trizol (Invitrogen Corporation, Carlsbad, CA, USA) according to manufacturer’s directions. Two µg of total RNA was reverse transcribed using random hexamer primers with SuperScript III Reverse Transcriptase (Invitrogen Corporation, Carlsbad, CA, USA) following the manufacturer’s directions. Gene expression levels were quantified using a CFX96 Real-Time System (Bio-Rad Laboratories, Hercules, CA, USA). *IL6* and *IL8* expression was normalized to *GAPDH* using the ΔΔCt method at each time point [30]. PCR primer sequences were as follows: IL-6-F, GGCACTGGCAGAAAACAACC; IL6-R, GCAAGTCTCCTCATTGAATCC; IL-8-F, AGAGTGATTGAGAGTGGACC; IL8-R, AACTTCTCCACAACCCTCTG; GAPDH-F, AGCCACATCGCTCAGACAC; GAPDH-R, CGCCCAATACGACCAAATCC.

### 2.4. Evaluation of DR4 and DR5 TRAIL Receptors by Flow Cytometry

The binding of mouse PE-conjugated DR4 and DR5 specific antibodies was utilized to quantitate the surface expression of DR4 and DR5 according to manufacturer’s guidelines (R&D systems, Minneapolis, MN, USA). Briefly, 1 × 10^6^ DU145 cells were harvested by treatment with PBS-0.5 mM EDTA for 10 min at room temperature, and then washed twice with flow cytometry staining buffer (FCSB). Cells were resuspended in FCSB, incubated with FcR blocking reagent for 15 min at room temperature followed by incubation with PE-conjugated DR4 or DR5 specific antibodies or PE-conjugated isotype control IgG for 45 min at 4 °C. Cells were washed twice with FCSB and examined by CyAn flow cytometry (Beckman Coulter, Brea, CA, USA).

### 2.5. Cytosolic and Nuclear Protein Extraction 

The cytosolic protein extraction procedure has been published [31]. Briefly, cells were suspended in ice-cold plasma membrane permeabilization buffer (200 µg/mL digitonin, 80mM KCl in PBS, with protease and phosphatase inhibitors) and incubated on ice for 5 min. The cell suspension was centrifuged at 800 × *g* for 5 min at 4 °C and supernatants were stored at −80 °C as cytosolic fractions for immunoblotting. The nuclear protein extraction was prepared using the NE-PER nuclear and cytoplasmic extraction reagents from Thermo Scientific (Rockford, IL, USA) according to the manufacturer’s directions.

### 2.6. Detection of Apoptosis by Flow Cytometry Analysis

Floating and attached cells released by trypsin/EDTA treatment were collected in RPMI 1640 medium with 10% fetal bovine serum, resuspended in PBS and stained with Annexin-V-FLUOS Staining Kit (Sigma-Aldrich Corporation, Saint Louis, MO, USA) for 20 min at room temperature. Cells were analyzed by flow cytometry as described previously.

### 2.7. Confocal Microscopy 

Treated and untreated cells were plated in Falcon four-well culture slides, fixed with 4% formaldehyde in PBS for 15 min and then rinsed three times with 1× PBS. The fixed cells were placed in blocking buffer (1 × PBS/5% normal serum/0.3% Triton × −100) for 60 min and then incubated with primary antibody at 4 °C overnight. After antibody removal, samples were incubated with secondary antibody for 1 h then covered with Prolong^®^ Gold Antifade Reagent with DAPI (Cell Signaling Technology, Beverly, MA, USA) and allowed to cure overnight at room temperature. Slides were stored at 4 °C and protected from light before imaging with a Zeiss LSM 700 Confocal Microscope (White Plains, NY, USA) and ZEN software (Version 2.5, Zeiss, White Plains, NY, USA).

### 2.8. Caspase 8 Activity Assay

Caspase 8 activity was detected by a colorimetric caspase 8 activity assay kit (Novus Biologicals, Centennial, CO, USA). Briefly, the cell lysate was incubated with IETD-pDN substrate at 37 °C for 1 h, as directed by the manufacturer. The absorbance at 405 nm was then read by a microplate reader (UVM340, ASYS Hitech GmbH, Austria).

### 2.9. Statistical Analyses

The statistical analyses were conducted using Student’s *t*-test (Microsoft Excel 2010). Differences were considered significant at *p* < 0.05.

## 3. Results

### 3.1. GlcN Induces ER Stress and Increases Cellular DR5 Expression in DU145 Cells

**Figure 1:**
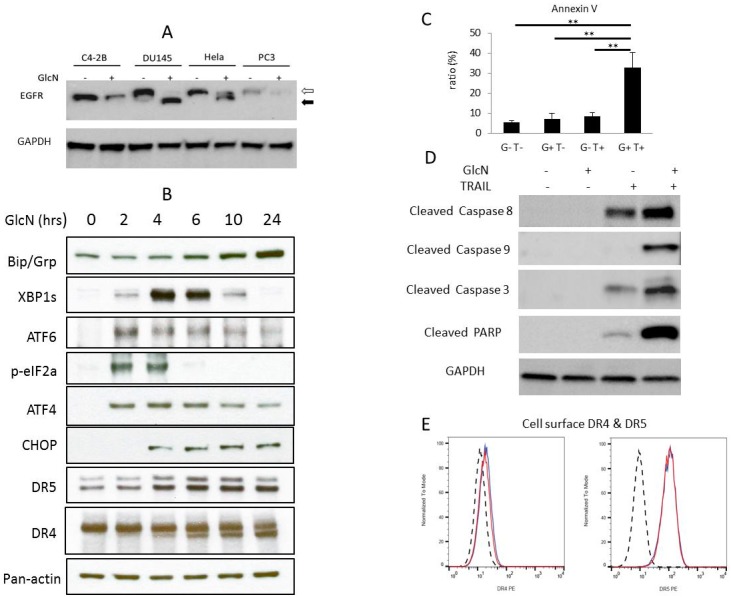
Glucosamine (GlcN) increases expression of multiple apoptosis effectors and synergizes tumor necrosis factor (TNF)-related apoptosis-inducing ligand (TRAIL)-induced apoptosis. (**A**) Sensitivity of different cell lines to GlcN-induced deglycosylation as measured by EGFR. C4-2B, DU145, HeLa and PC3 cells were cultured in the presence of 2 mM GlcN for 24 h. Whole-cell lysates were subjected to immunoblotting using an EGFR antibody. The open arrow indicates the N-glycosylated EGFR receptor and the filled arrow indicates the deglycosylated receptor. (**B**) DU145 cells were grown in 2 mM GlcN for the indicated time and whole-cell extracts were prepared and analyzed by immunoblotting using antibodies against DR4 and DR5 and multiple unfolded protein response (UPR) indicators (Bip, XBP1s, ATF6, p-EIF2a, ATF4, CHOP) and pan-actin. (**C**) Untreated (G–/T–) cells or cells treated with 2 mg/mL GlcN for 24 h (G+T–), 50 ng/mL TRAIL for 8 h (G–T+) or a combination of both GlcN for 24 h then TRAIL for 8 h (G+T+) were stained with annexin V/PI and then analyzed by flow cytometry analysis. Bars represent the mean value of three independent assays, **p < 0.01. (**D**) Whole-cell extracts were analyzed by immunoblotting with cleaved caspases 8, 9 and 3, PARP and GAPDH after treatment as in 1C. (**E**) Flow cytometry analysis of cell surface DR4/DR5 treated with 2 mM GlcN.

A number of cellular stress conditions including nutritional deprivation, hypoxia, alterations in protein glycosylation and disturbances of calcium flux can lead to the accumulation and aggregation of unfolded/misfolded proteins in the ER lumen inducing ER stress (unfolded protein response (UPR)) [32,33]. We first surveyed a variety of prostate cancer cell lines and HeLa cells to gauge the effects of GlcN-induced inhibition of N-glycosylation (hereafter described as deglycosylation) on EGFR, a known glycosylated protein [34]. Four cell lines were treated with 2 mM GlcN for 24 h and whole-cell extracts were analyzed by immunoblotting. Only DU145 and HeLa cells demonstrated substantial EGFR deglycosylation products after GlcN treatment (Figure 1A). Therefore, we elected to further study these two cells lines. To evaluate the effects of GlcN on ER stress, DU145 cells were treated with 2 mM GlcN for 2–24 h and whole-cell extracts were analyzed by immunoblotting as shown in Figure 1B. The ER stress sensor Bip/Grp78 initiates UPR via activation of three pathways: protein kinase PKR-like endoplasmic reticulum kinase (PERK), inositol-requiring protein 1a (IRE1) and activating transcription factor 6 (ATF6) [35]. The expression of Bip/Grp78 and the downstream protein targets of all three pathways of UPR (p-eIF2a, XBP1s and ATF6 50 kDa, respectively) were increased by the treatment of GlcN in a time-dependent manner with differing kinetics (Figure 1B). PERK phosphorylates eukaryotic initiation factor 2 (p-eIF2a) which inhibits global translation but promotes translation of selective mRNAs such as activating transcriptional factor 4 (*ATF4*). In turn, ATF4 activates the transcription of the ER stress responding gene *CHOP* which stimulates the expression of DR5 [32,36]. As shown in Figure 1B, ATF4, CHOP and DR5 protein levels increased after GlcN treatment. Taken together, we concluded that GlcN induced ER stress and enhanced the expression of DR5 in DU145 cells as previously demonstrated in other cell types [37].

In contrast to DR5, DR4 migrated as a single band in the absence of GlcN; in the presence of GlcN, a second faster migrating band appeared as a result of deglycosylation (Figure 1B) [15]. The total amount of DR4 protein (glycosylated and deglycosylated forms) did not change during the GlcN treatment as determined by band density quantification (data not shown). This result is consistent with the other N-glycosylation inhibitor, tunicamycin, which exhibits similar effects on DR4 deglycosylation in DU145 cells [38].

### 3.2. GlcN Potentiates TRAIL-Induced Apoptosis in DU145 Cells 

The higher expression of DR5 induced by GlcN could enhance TRAIL-induced apoptosis. To investigate this possibility, DU145 cells were pretreated with 2 mM GlcN for 24 h and then treated with TRAIL (50 ng/mL) for 8 h. The ability of GlcN to enhance apoptosis in combination with TRAIL (GlcN/TRAIL) was examined by flow cytometry analysis using annexin V and PI assays. Neither GlcN nor TRAIL alone induced the apoptotic response in cells; while treatment with GlcN/TRAIL induced apoptosis over 4-fold compared to untreated cells (*p* < 0.01) (Figure 1C). HeLa cells also demonstrated increased apoptosis to GlcN/TRAIL treatment albeit not in the same magnitude as DU145 cells, whereas neither PC3 nor C4-2B prostate cancer cells responded to GlcN/TRAIL treatment under the same conditions (Supplemental Data Figure S1A). To further characterize the nature of GlcN/TRAIL-induced apoptosis in DU145 cells, we quantified the cleavage of caspases 8, 9 and 3 and poly-ADP-ribose polymerase (PARP) by immunoblot analysis, as cleavage of these proteins are molecular indicators of apoptosis. Cells treated with GlcN alone did not demonstrate increased levels of cleaved caspases 8, 9 and 3 and PARP, while TRAIL alone increased caspase 8 and slightly increased the cleavage levels of caspase 3 and PARP (Figure 1D). However, treatment of cells with GlcN/TRAIL markedly increased TRAIL-induced cleavage of all three caspases as well as PARP. We observed similar behavior of the three caspases and PARP when deglycosylation-sensitive HeLa cells were treated with the GlcN/TRAIL combination under the same conditions (Supplemental Data Figure S1B). Taken together, our data indicated that GlcN, in a manner similar to tunicamycin, increased TRAIL-induced apoptosis by stimulating the activity of multiple caspases. The results support the contention that the activation of ER stress/UPR may be a useful approach to increase sensitivity of cells to TRAIL-induced apoptosis [12,14].

### 3.3. GlcN Does Not Alter the DR4 or DR5 Cell Surface Expression Level

Since the DR5 level was elevated in whole extracts analyzed by immunoblotting when cells were treated with GlcN, we asked if there was a concomitant increase in cell surface expression of DR5 since cell surface expression of DR5 initiates the apoptotic signaling cascade by binding to its ligand TRAIL. To test if GlcN treatment increased the cell surface expression of DR5, DU145 cells were treated with 2 mM GlcN for 24 h and analyzed by flow cytometry as described in Materials and Methods. Unexpectedly, GlcN treatment did not alter DR5 expression on the cell surface as no alteration in PE fluorescence intensity was detected with DR5 antibodies upon GlcN treatment (Figure 1E). DR4 surface expression was also examined and found to be very low regardless of GlcN treatment (Figure 1E). This indicated that even though GlcN/TRAIL treatment increased apoptosis, the mechanism was likely not through an increase in DR5 surface expression.

### 3.4. GlcN and TRAIL Treatment Overcome TRAIL-Resistance through Multiple Pathways

**Figure 2:**
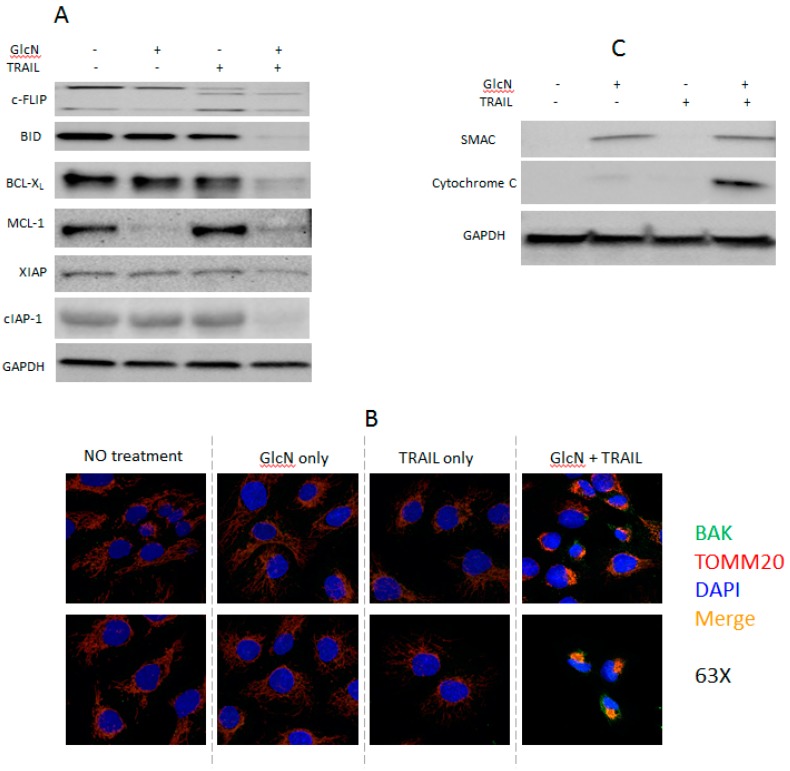
GlcN/TRAIL treatment in DU145 cells modulates both extrinsic and intrinsic apoptotic regulators. (**A**) Cells were treated as in Figure 1C and whole-cell extracts were prepared and analyzed by immunoblotting with antibodies against c-FLIP, BID, BCL-XL, MCL-1, XIAP, cIAP-1 and GAPDH. (**B**) Confocal imaging of DU145 cells treated the same as in A and stained with BAK (green), TOMM20 (red) and DAPI (blue), magnification 63×. (**C**) Cells were treated as described in A and cytosolic extracts were probed with antibodies against SMAC, cytochrome C and GAPDH.

Having found that GlcN/TRAIL treatment induced apoptosis but did not change the cell surface expression of DR5, we explored alternative mechanistic possibilities. We asked whether key signaling molecules involved in apoptosis and TRAIL resistance were altered by the GlcN/TRAIL combination [17,19,39]. cFLIP is a component of the DISC which plays a key role in the extrinsic apoptosis pathway and TRAIL resistance. GlcN alone did not change the expression of either long (L) or short (S) form of cFLIP while TRAIL treatment alone decreased cFLIP_L_, increased cFLIP_S_ and yielded a new intermediate band p43-cFLIP (Figure 2A). Compared to TRAIL alone, GlcN/TRAIL treatment further reduced expression of both cFLIP_L_ and cFLIP_S_ but did not increase p43-cFLIP.

TRAIL not only regulates extrinsic apoptosis but also can induce intrinsic apoptosis through BID. BID is a BH3-only protein which is a mediator between the extrinsic and intrinsic apoptotic pathways. Its cleavage by caspase 8 produces a truncated BID (tBID) which in turn activates BAK increasing mitochondrial permeabilization. A decrease of BID level was detected after GlcN/TRAIL treatment; the tBID cleavage product was not detected (Figure 2A). Since a decline of BID levels indicated the BID processing, we believed that the combination treatment cleaved BID to give tBID, which is known to suppress anti-apoptotic BCL-2 family members [40]. In addition, we also examined whether GlcN/TRAIL treatment altered the expression of BCL-2 family members [19,20]. The treatment dramatically suppressed the expression of MCL-1 and BCL-X_L_ (Figure 2A) [19,20]. GlcN alone had no effect on BCL-X_L_ expression but decreased MCL-1, suggesting that GlcN might utilize alternative mechanisms to regulate MCL-1 and BCL-X_L_ expression.

Since anti-apoptotic BCL-2 proteins suppress apoptosis via inhibition of BAK or BAX translocation from cytosol to the mitochondrial outer membrane, cellular localization of BAK upon GlcN/TRAIL treatment was investigated by confocal microscopy as described in Materials and Methods [2]. As shown in Figure 2B, BAK was clearly co-localized to the mitochondrial outer membrane with the outer membrane marker TOMM20 after GlcN/TRAIL treatment. As a result, pro-apoptotic cytochrome C and SMAC were released from the mitochondria into the cytoplasm by GlcN/TRAIL treatment (Figure 2C). We note that GlcN alone induced SMAC release from mitochondria, but not cytochrome C. Since GlcN alone did not lead to caspase cleavages and a significant increase in annexin V positive cells (Figure 1C,D), we concluded that GlcN-induced release of SMAC into the cytosol was insufficient to initiate apoptosis. BAX localization was not investigated, since DU145 cells are deficient in the protein [41]. Decreased expression of IAP family members are known to increase apoptosis [21,42], GlcN/TRAIL treatment decreased cIAP-1 significantly and XIAP slightly (Figure 2A). Collectively, these data demonstrated that GlcN/TRAIL treatment affected a variety of both extrinsic and intrinsic apoptotic factors.

### 3.5. GlcN Suppresses TRAIL-Activated NF-kB in DU145 Cells

**Figure 3:**
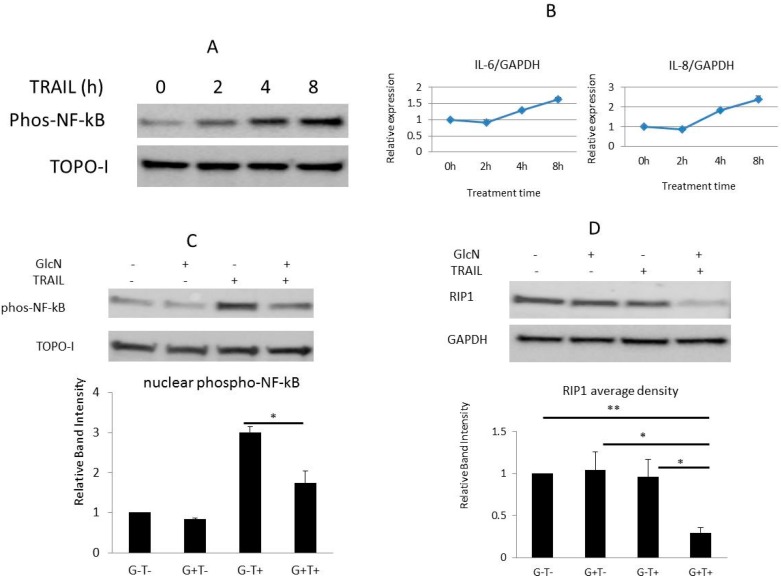
GlcN/TRAIL suppresses TRAIL-activated NF-kB signaling via inhibiting RIP1 and phosphorylation of NF-kB. (**A**) Cells were treated with TRAIL (50 ng/mL) for the designated time and nuclear extracts were prepared and analyzed by immunoblotting using antibodies against phospho-NF-kB and TOPO-1. (**B**) Cells were treated as in A, and total RNA was prepared for qRT-PCR analysis of *IL-6* and *IL-8* expression. Values represent the mean of two independent assays. (**C**) Cells were treated as in Figure 1C, nuclear extracts were prepared and analyzed by immunoblotting with antibodies against phospho-NF-kB, and TOPO-1. The bottom panel represents densitometric scanning of the results normalized by TOPO-1 levels. Bars represent the mean value of three independent assays, * *p* < 0.05. (**D**) Cells were treated as in Figure 1C, whole-cell extracts were prepared and analyzed by immunoblotting by using antibodies against RIP1 and GAPDH. The bottom panel represents densitometric scanning of the results normalized by GAPDH levels. Bars represent the mean value of three independent assays. * *p* < 0.05; ** *p* < 0.01.

In addition to apoptosis, TRAIL can simultaneously promote cellular survival through nuclear factor kappa B (NF-kB). To investigate whether GlcN is able to suppress the NF-kB signaling, DU145 cells were treated with TRAIL (50 ng/mL) for 2–8 h and nuclear extracts were analyzed by immunoblotting. As shown in Figure 3A, phosphorylation of NF-kB was stimulated in a time-dependent manner, a finding consistent with previous observations [43,44]. Furthermore, two well-known downstream targets of the NF-kB signaling, *IL6* and *Il-8* were up-regulated by TRAIL treatment (Figure 3B). These data showed that the NF-kB signaling was activated. Next the effect of GlcN on NF-kB was examined. Our data demonstrated that GlcN alone had no effect on the basal NF-kB activity, while combined GlcN/TRAIL treatment suppressed (*p* < 0.05) the TRAIL-stimulated NF-kB activation (Figure 3C). Since the presence of RIP1 is required for the TRAIL-induced NF-kB activation, we tested if GlcN/TRAIL treatment suppressed RIP1 expression [45]. As shown in Figure 3D, RIP1 levels were significantly reduced with GlcN/TRAIL treatment while TRAIL alone did not change RIP1 expression. Taken together, our data support that the GlcN/TRAIL combination suppresses the TRAIL-stimulated NF-kB activation and that GlcN could shift the balance toward apoptosis.

### 3.6. GlcN Enhanced TRAIL-Induced Apoptosis is Caspase 8-Dependent

**Figure 4:**
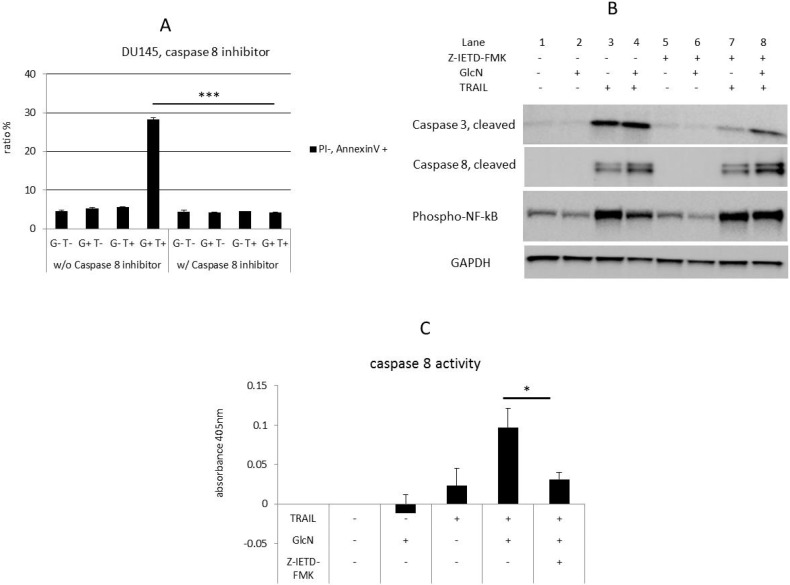
Suppression of caspase 8 activity leads to the rescue of GlcN/TRAIL-induced apoptotic cell death. DU145 cells were treated as in Figure 1C in the absence (-) or presence (+) of the caspase 8 inhibitor Z-IETD-FMK. (**A**) After treatment, cells were stained with annexin V/PI and then analyzed by flow cytometry analysis. Bars represent the mean value of three independent assays, *** *p* < 0.001. (**B**) Whole-cell extracts were prepared and analyzed by immunoblotting with antibodies against cleaved caspases 3 and 8, and phospho-NF-kB. (**C**) Caspase 8 activity was measured. Bars represent the mean value of three independent assays, * *p* < 0.05.

Since both TRAIL-induced apoptotic and survival functions are mediated by caspase 8 [23,46], we hypothesized that the GlcN/TRAIL combination would neither induce apoptosis nor suppress NF-kB if caspase 8 activity was blocked. To test this, the specific caspase 8 inhibitor Z-IETD-FMK was utilized to ablate its activity. GlcN/TRAIL-induced apoptosis was blocked by Z-IETD-FMK treatment as shown by flow cytometer analysis with annexin V (Figure 4A, *p* < 0.001). Surprisingly, the Z-IETD-FMK increased caspase 8 levels in the presence of GlcN/TRAIL, but reduced caspase 3, a downstream target of caspase 8 (Figure 4B, Lane 4 vs. Lane 8). Measurement of caspase 8 enzymatic activity showed that Z-IETD-FMK strongly suppressed the GlcN/TRAIL-induced caspase 8 activity (Figure 4C, *p* < 0.05). In addition, Z-IETD-FMK restored previously suppressed NF-kB levels by GlcN/TRAIL treatment (Figure 4B, Lane 4 vs. Lane 8), whereas NF-kB was unaffected by Z-IETD-FMK in the presence of TRAIL alone (Figure 4B, Lane 3 vs. Lane 7). This suggested that the NF-kB activation by TRAIL was independent of caspase 8 enzymatic activity. On the contrary, NF-kB suppression through the GlcN/TRAIL treatment required caspase 8 enzymatic activity. Collectively, these data support the role of caspase 8 as a key regulator to induce GlcN/TRAIL apoptosis and suppress NF-kB activity.

## 4. Discussion

Weak responses of human tumors to TRAIL prompted us to search for approaches to increase the efficacy of TRAIL therapy [6]. ER stress leads to elevated expression of DR5 which stimulates TRAIL-induced apoptotic activity in cancer cells [8,47]. *DR5* expression is regulated by transcription factors such as CHOP which is activated by multiple ER stressors including the N-glycosylation inhibitor tunicamycin, the calcium ATPase inhibitor thapsigargin, the glycolysis inhibitor 2-deoxy-D-glucose (2-DG) and the proteasome inhibitor MG132 [9,48,49,50,51]. We demonstrated that GlcN increased DR5 expression in whole-cell extracts and enhanced TRAIL-induced apoptosis in DU145 cells (Figure 1C). However, when we examined cell surface expression, we did not observe an associated increase of DR5. These data suggested that GlcN/TRAIL treatment induced apoptosis without alteration in cell surface presentation of DR5. Prior work showed that nuclear DR5 was associated with TRAIL-resistance [52]. However, a survey of subcellular compartmentalization indicated that GlcN did not increase nuclear DR5 levels (Supplementary Data Figure S1C). Very recently, Liang et al. have reported that N-acetyl-glucosamine (GlcNAc) sensitizes A549 cells to TRAIL-induced apoptosis thorough the formation of DR5 clusters on the plasma membrane, thereby increasing DISC formation which triggers apoptosis [53]. Since GlcN is acetylated to give GlcNAc in cells and activates the hexosamine signaling pathway, it might be possible that the increased DR5 protein by GlcN treatment could localize to plasma membrane in a similar manner to GlcNAc [54]. Furthermore, GlcN could O-glycosylate DR5 like GlcNAc, which increases tumor-cell sensitivity to TRAIL [55]. Thus, the cellular localization and functional consequences of GlcN-induced DR5 deserve further investigation. 

Since elevated cell surface DR5 expression was not detected, we tested several well-known molecules associated with TRAIL-resistance. One such protein is cFLIP, a master anti-apoptotic regulator, which suppresses TRAIL-induced apoptosis and has two isoforms, cFLIP_L_ (50 kDa) and cFLIP_S_ (26 kDa) [17,56]. cFLIP_L_ and procaspase 8 are structurally similar and form a heterodimer that leads to the inhibition of apoptosis. This interaction also leads to the activation of NF-kB [17]. Unlike cFLIP_L_, cFLIP_S_ does not possess C-terminal caspase-like domains; therefore, it inhibits caspase 8 cleavage and apoptosis [17,57]. TRAIL treatment led to a new p43-cFLIP (Figure 2A) fragment, derived from the cleavage of cFLIP_L_ after the formation of a heterodimer with procaspase 8; yet the truncated form retains cFLIP_L_ function [58,59]. Overexpression of cFLIP is clearly related to the increased resistance to TRAIL treatment [17]. Compared to TRAIL alone, GlcN/TRAIL treatment decreased cFLIP_L_ and cFLIP_S_, but did not change p43-cFLIP levels. The reduction of cFLIP_L_ and cFLIP_S_ may contribute to the elevated apoptotic effects observed with GlcN/TRAIL treatment. 

BCL-2 family proteins also play important roles in the regulation of apoptosis. Our results showed that GlcN/TRAIL combination reduced the levels of anti-apoptotic proteins BCL-X_L_ and MCL-1. This reduction facilitates the translocation of BAK to the outer mitochondrial membrane from the cytosol [2,20]. Consistent with this observation, our confocal microscopy data revealed that GlcN/TRAIL treatment induced BAK translocation to the outer mitochondrial membrane, while neither GlcN nor TRAIL alone did (Figure 2B). The translocation released SMAC and cytochrome C from mitochondria to the cytosol. SMAC antagonizes XIAP by competing with caspase 9 for binding BIR3 domain of XIAP and cytochrome C forms apoptosome, both of which activate caspase 9 [60]. In addition, GlcN/TRAIL treatment suppressed anti-apoptotic XIAP and cIAP-1 expression levels (Figure 2A). Our data demonstrated that GlcN/TRAIL treatment targets multiple molecules associated with TRAIL-resistance, such as cFLIP, BID, MCL-1, BCL-X_L_, XIAP and cIAP-1. 

The caspase 8 specific inhibitor Z-IETD-FMK suppressed apoptosis induced by GlcN/TRAIL combination as expected, since caspase 8 enzymatic activity is required for apoptosis. This inhibitor suppresses caspase 8 enzymatic activity by binding to its catalytic domain and therefore does not affect its non-enzymatic activity, i.e., its role as a scaffold for the assembly of a pro-inflammatory complex to activate NF-kB [61]. Z-IETD-FMK increased caspase 8 under the conditions of GlcN/TRAIL treatment (Figure 4B, Lane 4 vs. Lane 8), whereas its enzymatic activity was suppressed (Figure 4C). Consistent with this, Z-IETD-FMK increased the phosphorylated NF-kB levels which were downregulated by GlcN/TRAIL treatment (Figure 4B, Lane 4 vs. Lane 8).

**Figure 5:**
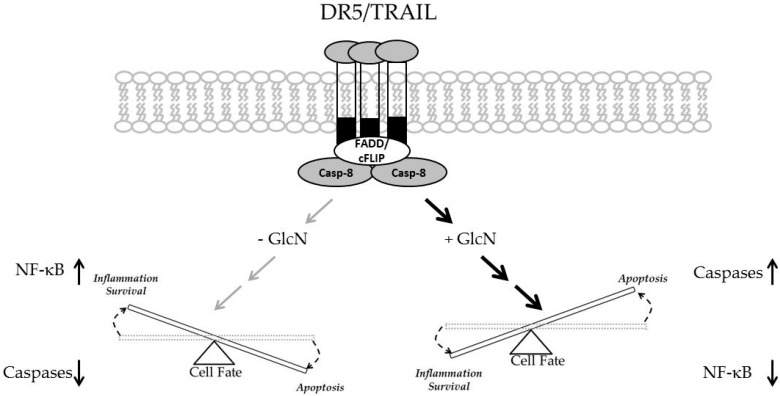
GlcN increases the flux toward apoptosis versus inflammation (anti-apoptotic) in combination treatment with TRAIL. The addition of GlcN/TRAIL increases overall caspase levels and activates molecules associated with the intrinsic apoptotic pathway (i.e., SMAC and cytochrome C). GlcN/TRAIL also reduces inflammatory pathways by reducing NF-kB levels which are typically stimulated by TRAIL treatment alone.

TRAIL is a double-edged sword in terms of its pro-apoptotic and anti-apoptotic properties [22]. GlcN/TRAIL combination treatment shifts the balance between inflammatory and apoptotic pathways (Figure 5). On one hand TRAIL can activate the apoptotic process through the formation of DISC upon binding to its receptors. On the other hand TRAIL can activate anti-apoptotic processes mainly through the activation of the NF-kB signaling. Our results demonstrated that in the presence of GlcN, TRAIL increased the apoptotic commitment while at the same time suppressing TRAIL-induced NF-kB activation.

## Supplementary Materials

The following are available online at https://www.mdpi.com/2305-6320/6/4/104/s1, Figure S1. Cells insensitive to N-linked deglycosylation fail to respond to GlcN/TRAIL-induced apoptosis.
