# Supplementary material for: Glucosamine Enhances TRAIL-Induced Apoptosis in the Prostate Cancer Cell Line DU145
Source: Medicines (Basel). 2019 Oct 15;6(4):104. doi: 10.3390/medicines6040104 (PMC6963486; doi:10.3390/medicines6040104)
Supplement: Supplementary file 1 [file medicines-06-00104-s001.pdf]

Supplementary Materials: Glucosamine Enhances TRAIL-Induced Apoptosis in the Prostate Cancer Cell Line DU145

Chao Sun, Viktor Chesnokov, Garrett Larson and Keiichi Itakura

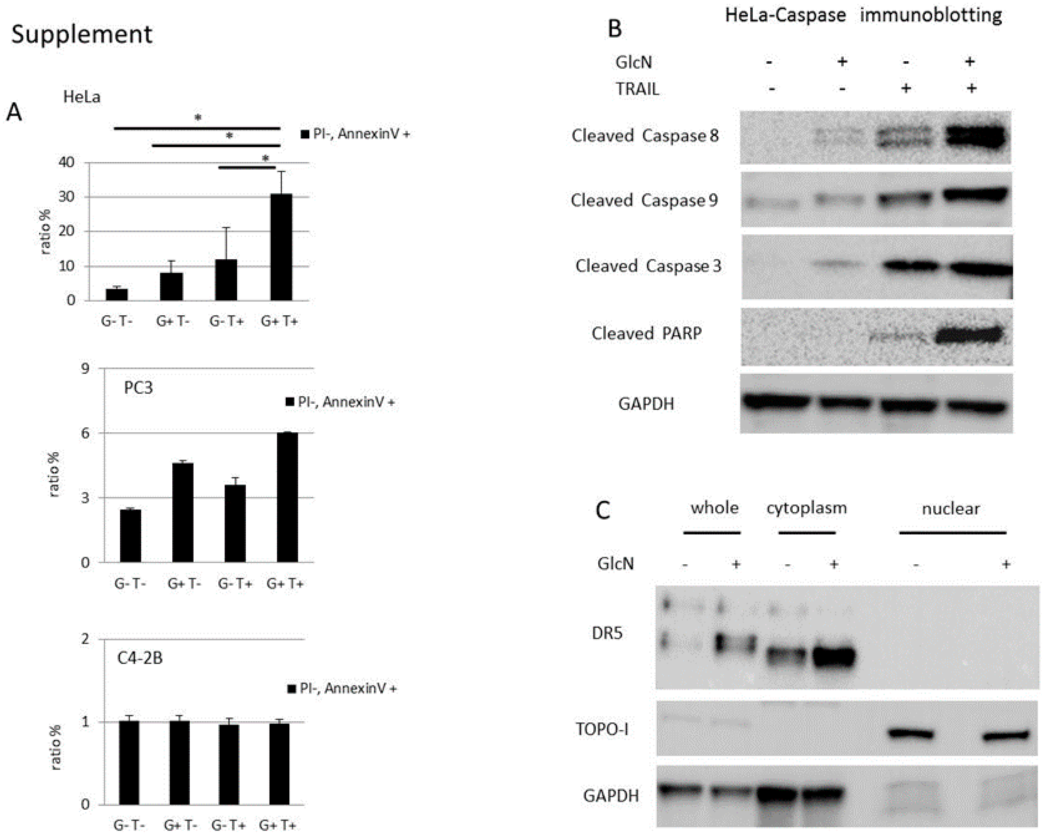

Figure S1. Cells insensitive to N-linked deglycosylation fail to respond to GlcN/TRAIL-induced apoptosis. (**A**) HeLa, PC3 or C4-2B cells were treated as in Figure 1C. After treatment, cells were stained with annexin V/PI and then analyzed by flow cytometry. Bars represent the mean value of two independent assays, $*p < 0.05$. (**B**) Immunoblot analysis of whole-cell HeLa extracts cultured as described in Figure 1C and probed with antibodies against the cleaved forms of caspases 8, 9 and 3, PARP and GAPDH. (**C**) DU145 cells were treated with 2 mM GlcN for 24 h and protein extracts from whole-cell, cytoplasmic or nuclear fractions were subjected to immunoblotting using a DR5 antibody. TOPO-1 and GAPDH were used as loading controls for nuclear and whole-cell/cytoplasmic extracts, respectively.
